# Assessment of Different Indices as Predictors of Difficult Airway in Obese Patients

**DOI:** 10.7759/cureus.55005

**Published:** 2024-02-26

**Authors:** Varun Sharma, Himanshu P Yadav, Abhishek Prakash, Namrata Yadav, Mukesh Kumar, Haider Abbas

**Affiliations:** 1 Cardiac Anesthesia, Chandan Hospital, Lucknow, IND; 2 Anesthesiology, Kalyan Singh Superspeciality Cancer Institute, Lucknow, IND; 3 Critical Care Medicine, Apollomedics Super Specialty Hospitals, Lucknow, IND; 4 Respiratory Medicine, Dr. Ram Manohar Lohia Institute of Medical Sciences, Lucknow, IND; 5 Emergency Medicine, King George's Medical University, Lucknow, IND

**Keywords:** body mass index: bmi, thyro-mental distance, skin fold thickness, difficult airway management, obesity

## Abstract

Introduction

Difficulties with tracheal intubation contribute to the morbidity and mortality associated with anesthesia. Suggested predictors for difficult airway include, history of obstructive sleep apnea, high Mallampati score, elderly, male, short neck, and high Wilson score. However, none of these has high diagnostic accuracy particularly in obese population. Parameters used to quantify obesity such as Body Mass Index(BMI), mid arm circumference, skin fold thickness, etc. have not been used as independent predictors of difficult airway. This study has been designed to evaluate the accuracy of commonly used tools to assess difficult airway and to test other obesity markers as scale for assessing difficult airway i.e. Bag mask ventilation grade ≥3 or Cormack-Lehane Grade≥3 on Direct Laryngoscopy or number of intubation attempts ≥3.

Aim

To assess BMI, Mallampati grading, Neck circumference and Thyromental distance as predictors of difficult airway in obese patients and to validate neck circumference to thyromental distance ratio and skin fold thickness as a tool for assessment of difficult airway (Bag mask ventilation grade ≥3 or Cormack-Lehane Grade≥3 on Direct Laryngoscopy or number of intubation attempts ≥3.) in obese patients.

Methods

This prospective observational study was carried out on 51 obese patients (as per BMI) of ASA grade II, either sex, aged 23 to 57 years posted for elective surgery under general anesthesia with endotracheal intubation. After subjective assessment of difficult airway following data sets and variables were obtained - sex, weight, height, body mass index (BMI), Modified Mallampati class (MPG), Cormack-Lehane (CL) grade, adequacy of bag mask ventilation (BMV), number of endotracheal intubation attempts, patient’s neck circumference (NC), thyromental distance (TMD), waist-hip ratio (WHR) and Skin fold thickness. The observations made during the study were statistically analyzed and correlated as predictors for difficult airway.

Result

Correlation of MPG to CL-grade (r-0.41, p-0.003), BMV (r-0.31, p-0.028) & No. of intubation attempts (r-0.37, p-0.007) was mild & statistically significant. Correlation of Neck Circumference with CL-grade (r-0.57, p-0.000), & No. of intubation attempts (r-0.62, p-0.000), found moderate & statistically significant, & with BMV was mild and statistically significant (r-0.48, p-0.000). Correlation of Thyromental Distance to CL-grade (r-0.65, p-0.000), BMV (r-0.70, p-0.000) & No. of intubation attempts (r-0.61, p-0.000) was moderate & statistically significant. Correlation of BMI to CL-grade (r-0.11, p-0.428), BMV (r-0.04, p-0.757) & No. of intubation attempts (r-0.16, p-0.257) was weak & not significant.

Skin Fold Thickness showed no significant association with Difficult airway i.e., CL Grade (p-0.478), BMV (p-0.101), and No. of intubation attempts (p-0.143). Correlation of NC/TMD ratio with BMV (r-0.74, p-0.000), CL-grade (r-0.76, p-0.000), & No. of intubation attempts (r-0.77, p-0.000) was moderate & statistically significant.

Conclusion

NC, TMD and NC/TMD Ratio depicted a close association with airway difficulty in obese patients. Obesity grade is a risk factor for difficult airway but predictors of obesity including Skin Fold Thickness, individually did not show association with difficult airway (small sample size may be a limiting factor). None of the commonly performed tests alone has proven to be adequate in predicting difficult intubation in the obese population.

## Introduction

Airway management is a major responsibility for anesthesiologists. Difficulties with tracheal intubation significantly contribute to the morbidity and mortality associated with anesthesia [1]. Identifying situations and patients at frequent risk for airway management problems is a key to optimal care and has been the focus of numerous publications. Several reviews have reported that endotracheal intubation is more difficult in obese than in lean patients. However, this assertion remains debated because other studies have found no evidence that tracheal intubation is more difficult in obese than in lean individuals. One of the reasons for these discrepancies is the lack of consensus on the definition of the term “difficult intubation,” which varies between authors [ 2,3].

A “difficult airway” has been defined as a clinical situation in which a conventionally trained anaesthesiologist experiences problems with mask ventilation, or tracheal intubation, or both. The incidence of difficult laryngoscopy and tracheal intubation is unknown, but it may be as frequent as 7.5% in the normal surgical population. The medical literature on this subject is confusing because a poor laryngoscopic view does not always equate with a difficult airway [4].

Difficult airway management is one of the principal challenges faced by anesthesiologists in their routine practice [ 5]. Data published by the American Society of Anesthesiologists (ASA) shows that, despite the decline registered over recent decades, adverse respiratory events were involved in 34% of all lawsuit claims in the 1990s [ 6]. Difficult intubation, inadequate ventilation, and esophageal intubation were the principal factors responsible for death or brain damage.

Opinions are divided as to whether or not tracheal intubation is more difficult in obese patients and available data are inconclusive. Many attempts have been made to develop reliable predictors for difficult intubation or difficult laryngoscopy.

Suggested predictors for difficult intubation include a history of obstructive sleep apnoea, high Mallampati score, elderly, male, short neck, and high Wilson score. The Mallampati score or increasing neck circumference (NC) is reported to be associated with difficult intubation, especially in obese patients. Other parameters used to quantify obesity such as waist-hip ratio, mid-arm circumference, skin fold thickness, BMI, etc. have not been used as individual predictors of difficult airway. This study has been designed to evaluate the accuracy of commonly used tools to assess difficult airways and to test the other obesity markers as a scale for assessing difficult airways (bag mask ventilation grade ≥3 or Cormack-Lehane Grade≥3 on Direct Laryngoscopy or the number of intubation attempts ≥3).

## Materials and methods

This prospective observational study was carried out in Gandhi Memorial and Associated Hospitals, King George's Medical University, Lucknow, on patients of age more than 20 years posted for elective or emergency surgeries requiring endotracheal intubation under direct laryngoscopy for general anesthesia. Patients of age <20 years, or having congenital facial anomalies, temporomandibular joint (TMJ) ankylosis, traumatic facial injuries, facial burns, pregnant, patients with cognitive deficiencies, beard, moderate-to-severe ascites, and cervical spine diseases were excluded from the study. Written and informed consent was taken from the patients falling in the inclusion criteria; patients who denied the consent were not included in the study. A difficult airway is defined as bag mask ventilation grade ≥3 or Cormack-Lehane Grade≥3 on Direct Laryngoscopy or the number of intubation attempts ≥3 in our study. Following selection, a pre-anesthetic evaluation was performed by an anesthesiologist and/or a resident anesthesiologist.

At the time of airway assessment, the following data sets were obtained.

Mallampati score assessment

The patient’s modified Mallampati score assessment was performed with the patient sitting straight with mouth open and protruding tongue maximally without speaking anything (Table 1).

**Table 1:** Modified Mallampati Classification Mallampati et al. [7]

Mallampati Grade	Structures Visualised
Class I	soft palate, uvula and tonsillar pillars are visible
Class II	soft palate and uvula, are visible.
Class III	only soft palate and base of the uvula are visible.
Class IV	only hard palate is visible.

BMI

The patient’s BMI is calculated as: Body mass index (Quetlet’s index) = Weight (kg)/Height² (m) 

Cut-off points proposed by the WHO Expert Committee for the classification of obesity on the basis of BMI are given in Table 2.

**Table 2:** WHO classification of obesity James et al. [8]

BMI (kg/m^2)^	Classification
< 18.5	Underweight
18.5-24.9	Normal range
25.0-29.9	Pre-obese
30.0-34.9	Obese Grade 1
35.0-39.9	Obese Grade 2
> 40.0	Obese Grade 3

Obesity metrics

The patient’s neck circumference was measured with standard technique: midway of the neck, between the mid-cervical spine and mid-anterior neck, using non-stretchable plastic tape with the subject sitting/standing upright, at the superior border of the measuring tape was placed just below the laryngeal prominence and applied perpendicular to the long axis of the neck.

The thyromental distance was measured with non-stretchable plastic tape from the thyroid notch of the tip of the jaw with the head in the extended position.

The waist-hip ratio (WHR) was measured by using a non-stretchable measuring tape. (Waist circumference was measured at the midpoint between the lower border of the rib cage and the iliac crest, and hip circumference was measured at the widest part of the buttocks.)

Skin fold thickness

Skin fold thickness was measured by using skin calipers (digital body fat calipers with a range of 0-50 mm, resolution 0.1 mm, and accuracy 0.2 mm). All skin fold measurements were taken from the supra-iliac region (intersection of a line joining the anterior (front) part of the axilla (armpit), and a horizontal line at the level of the iliac crest).

In the operation theatre, patients were placed in the dorsal decubitus position and in the sniffing position, which consists of flexing the neck and then extending the head. A pillow was used to support the head to add sufficient height so as to ensure that the external auditory meatus and the sternal notch were aligned horizontally.

Standard monitoring and protocol were used for anesthesia, after premedication (injection of midazolam 0.02 mg/kg) and pre-oxygenation (for 3 minutes). General anesthesia was induced with an intravenous inducing agent (injection propofol 2 mg/kg) till loss of verbal response. The adequacy of the bag mask ventilation (BMV) was assessed using a 4-point grading. The four grades of ventilation used in the study are: 1 - ventilated without oral airway; 2 - ventilated with oral airway/adjuvant; 3 - difficult ventilation characterized as unstable, inadequate, or requiring two providers despite oral airway/adjuvant; 4 - unable to ventilate despite multiple providers or adjuvants. A neuromuscular blocking drug was then administered. After the latency period (1 minute for succinylcholine (1.5 mg/kg) and 5 minutes for vecuronium (0.1 mg/kg)/atracurium(0.5 mg/kg)), laryngoscopy performed using a conventional laryngoscope with a Macintosh blade # 3, 4 or 5, and Cormack-Lehane grade (Grade I: if most of the glottis, Grade II: if only the posterior extremity of the glottis is visible, Grade III: if no part of the glottis can be seen but only the epiglottis, Grade IV: if not even the epiglottis can be exposed.) noted down on the basis of laryngoscopic view [9].

Endotracheal intubation by direct laryngoscopy was then performed for all patients, with the number of attempts required until successful intubation, or the impossibility of endotracheal intubation, recorded.

Data concerning sex, weight, height, and BMI were recorded along with the following variables: the modified Mallampati class, the Cormack-Lehane grade, and the number of endotracheal intubation attempts. For the purpose of data analysis, the patients were subdivided into groups for each index: Mallampati I/II or Mallampati III/IV; Cormack-Lehane grade I/II or III/IV; the number of attempts at intubation <3 or ≥3 or failed intubation (there was no failed intubation).

The observations made during the study were then statistically analyzed and correlated as predictors for difficult airways.

Data management and analysis

Data was entered in a Microsoft Excel sheet (Microsoft Corporation, Redmond, USA). The confidentiality of each study participant was maintained throughout the study by using a non-medical ID number. The data was analyzed using SPSS version 24.0 (IBM Corp., Armonk, USA). A descriptive summary using frequencies, percentages, graphs, mean, median (IQR), and standard deviation was used to present the study results. Probability (p) was calculated to test statistical significance at the 5% level of significance. Categorical variables were analyzed using the chi-square test. Continuous variables were calculated using an independent t-test. Mann-Whitney U test was used to compare the differences in medians between different groups. For positive correlation and negative correlation, the Pearson correlation coefficient was used with r < 0.3 as weak or no correlation, r = 0.3 to 0.5 as mild correlation, r = 0.5 to 0.7 as moderate correlation, r = 0.7 to 0.9 as strong correlation, and r > 0.9 as very strong to perfect correlation; the level of significance was represented as "p" where p > 0.05 = not significant, p <0.05 = significant, p <0.01 = highly significant, p <0.001 = very highly significant.

## Results

A total of 51 patients who gave their consent were included in the study. The range of age of the study population was 23-57 years, median age was 41 years, and mean age was 40.75+8.62 years. The most common age group was 40-49 years (43.14%) followed by 30-39 years (31.37%) while the proportion of patients aged 50-59 (13.73%) and 20-29 years (11.76%) was lower than the above. Out of 51 patients included in the study, 30 (58.82%) were male and the rest, 21 (41.18%), were female (Tables 3, 4).

**Table 3:** Distribution of study samples according to age (years) Range: 23-57 years; Median: 41 years; Mean±SD = 40.75+8.62 years

Age Group	Number of cases	Percentage
20-29	6	11.76
30-39	16	31.37
40-49	22	43.14
50-59	7	13.73
Total	51	100.00

**Table 4:** Anthropometric variables in the study population (N=51)

	Min.	Max.	Median	Mean	SD
Height (cms)	100.00	180.00	160.00	156.00	13.00
Weight (kg)	65.00	110.00	85.00	86.51	11.65
BMI (kg/m^2^)	26.50	48.80	34.60	35.13	3.96

The most common obesity grade in our study population was Grade I (45.10%) followed by Grade II (39.22%), while Pre-obese and Grade III obese, each was 7.84% of the population (Table 5).

**Table 5:** Distribution of study samples according to grade of obesity Range: 26.50-48.80 kg/m2; Median: 34.60 kg/m2; Mean±SD = 35.13+3.96 kg/m2

Grade of Obesity	BMI	Number of cases	Percentage
Pre-obese	25.0-29.99 kg/m^2^	4	7.84
Obese Grade I	30-34.99 kg/m^2^	23	45.10
Obese Grade II	35-39.99 kg/m^2^	20	39.22
Obese Grade III	>=40 kg/m^2^	4	7.84
Total		51	100.00

The WHR of all patients in the study indicated a very high risk of obesity-related diseases (Table 6).

**Table 6:** Obesity variables in study population (N=51) NC: neck circumference; TMD: thyromental distance

	Min.	Max.	Median	Mean	SD
Neck circumference (NC) (cms)	35.00	49.00	40.00	40.80	3.76
Thyromental distance (TMD) (cm)	6.00	11.00	9.00	8.97	1.20
NC/TMD ratio	3.50	7.30	4.20	4.59	0.89
Skin fold thickness (mm)	21.50	42.50	30.60	31.27	4.08
Waist (cm)	89.00	138.00	102.00	102.35	9.66
Hip (cm)	80.00	110.00	88.00	89.41	7.66
Waist-Hip ratio	1.04	1.27	1.13	1.14	0.06

The Mallampati Grade of the study population ranged from 1-4; the median grade was 3 and the mean was 2.76+0.55. Cormack-Lehane (CL) Grade of the study population ranged from 1-4; the median grade was 2 and the mean was 2.29+0.76. The bag mask ventilation (BMV) grade of the study population ranged from 1 to 3; the median grade was 1 and the mean was 1.37+0.63. The number of intubation attempts in the study population ranged from 1 to 4; the median number of attempts was 1 while the mean number of attempts was 1.51+0.88 (Table 7).

**Table 7:** Parameters of difficulty grades in study population (N=51)

	Min.	Max.	Median	Mean	SD
Mallampati Grade (MPG)	1	4	3	2.76	0.55
Cormack-Lehane Grade (CL)	1	4	2	2.29	0.76
Bag-mask ventilation (BMV)	1	3	1	1.37	0.63
Number of intubation attempts	1	4	1	1.51	0.88

The airway was defined to be difficult when the patient had Cormack-Lehane Grade ≥3, bag mask ventilation grade ≥3, and the number of intubation attempts ≥3. The incidence of difficult airways in the present study was 7.84% only (Table 8).

**Table 8:** Incidence of difficult airway

	Number of cases	Percentage
Normal Airway	47	92.16
Difficult Airway	4	7.84
Total	51	100.00

Out of the 51 patients, 16 (31.37%) had CL grade <3 and the rest 35 (68.63%) had CL-grade 1-2. The proportion of patients aged 30-39 years was higher having low CL grade (34.29%) as compared to those having high CL grade (25.00%). In the rest of the age groups, the proportion of patients with a high CL grade was higher than those having Low CL-grade, but the difference was not found to be statistically significant (p=0.863). Though proportion of males was higher in low CL grade (62.86%) as compared to high CL grade (50.00%), and proportion of females was higher in high CL grade (50.0%) as compared to low CL grade (37.14%), but difference in the CL grades of the male and female populations was not found to be statistically significant (p=0.387) (Table 9).

**Table 9:** Association of Cormack-Lehane (CL) grade with demographic variables Low CL Grade : Grade 1 & Grade 2; High CL Grade >3 χ2- Chi Square

Variables	Total	Low CL Grade (n=35)		High CL Grade (n=16)		Statistical Significance	
		No.	%	No.	%	χ^2^	P
Age Group (years)							
20-29	6	4	11.43	2	12.50	0.743	0.863
30-39	16	12	34.29	4	25.00		
40-49	22	15	42.86	7	43.75		
50-59	7	4	11.43	3	18.75		
Gender							
Female	21	13	37.14	8	50.00	0.749	0.387
Male	30	22	62.86	8	50.00		

The proportion of patients was higher in low CL grade as compared to high CL grade in preobese (8.57% vs. 6.25%), obese grade I (48.57% vs. 37.5%), obese grade II (42.86% vs. 31.25%) while the proportion of patients was higher in high CL grade as compared to low CL grade in obese grade III (25.0% vs. 0.0%). Difference in CL grade in different grades of obesity was found to be statistically significant (p=0.023) (Table 10).

**Table 10:** Association of Cormack-Lehane grade with obesity grade χ2- Chi square

Obesity Grade	Total	Low CL Grade (n=35)		High CL Grade (n=16)		Statistical Significance	
		No.	%	No.	%	χ^2^	P
Pre-obese	4	3	8.57	1	6.25	9.501	0.023
Obese Grade I	23	17	48.57	6	37.50		
Obese Grade II	20	15	42.86	5	31.25		
Obese Grade III	4	0	0.00	4	25.00		

Though the mean age, weight, and BMI of patients of high CL grade were found to be higher than that of low CL grade and vice-versa for height, the difference in the mean value of the above variables of high CL grade and low CL grade patients was not found to be statistically significant (p>0.05). Mean values of all other variables except thyromental distance of high CL-grade patients were found to be higher than that of low CL grade but the difference in mean values of patients with Low CL-grade and High CL-grade was found to be statistically significant for neck circumference (44.56+3.72 vs. 39.09+2.23 cm), thyromental distance (7.84+1.08 vs. 9.49+0.85 cm), NC/TMD ratio (5.69+0.72 vs. 4.09+0.34) and MP Grade (2.63+0.49 vs. 3.06+0.57) only (Table 11).

**Table 11:** Association of Cormack-Lehane grade and patient characteristics affecting obesity *Statistical analysis performed using Mann-Whitney U test NC/TMD: neck circumference/thyromental distance ratio; W/H ratio: waist-hip ratio; MG grade: Mallampati grade

	Low CL Grade (n=35)		High CL Grade (n=16)		Statistical significance	
	Mean	SD	Mean	SD	't'/Z*	'p'
Age (yrs)	40.31	8.34	41.69	9.41	-0.524	0.603
Weight (kg)	84.94	11.99	89.94	10.38	-1.436	0.157
Height (mt)	1.56	0.10	1.54	0.17	0.512	0.611
BMI (kg/m^2^)	34.50	3.18	36.49	5.15	-1.695	0.096
Neck circumference (cm)	39.09	2.23	44.56	3.72	-6.550	<0.001
Thyromental distance (cm)	9.49	0.85	7.84	1.08	5.870	<0.001
NC/TMD ratio	4.09	0.34	5.69	0.72	-10.830	<0.001
Skin fold thickness (mm)	30.89	3.83	32.11	4.59	-0.992	0.326
Waist(cm)	101.49	9.75	104.25	9.46	-0.948	0.348
Hip (cm)	89.40	7.64	89.44	7.95	-0.016	0.987
W/H ratio	1.13	0.06	1.16	0.05	-1.818	0.075
MP Grade*	2.63	0.49	3.06	0.57	-2.468	0.014

Out of 51 patients, only four (7.84%) had high BMV, i.e. Grade >3, and the rest 47 (92.16%) had BMV Grade 1-2. The difference in age of patients with High BMV and Low BMV was not found to be statistically significant (p=0.718). The proportion of females was higher in high BMV grade (100.0%) as compared to low BMV grade (36.17%) and the proportion of males was higher in low BMV (63.83%) as compared to high BMV (0.00%). The difference in the grades of BMV of males and females was found to be statistically significant (p=0.013) (Table 12).

**Table 12:** Association of bag mask ventilation (BMV) grade with demographic variables Low BMV Grade: Grade 1 &Grade 2; High BMV Score >3 χ2 - Chi Square

Variables	Total	Low BMV Grade (n=47)		High BMV Grade (n=4)		Statistical Significance	
		No.	%	No.	%	χ^2^	P
Age Group (years)							
20-29	6	5	10.64	1	25.00	1.346	0.718
30-39	16	15	31.91	1	25.00		
40-49	22	20	42.55	2	50.00		
50-59	7	7	14.89	0	0.00		
Gender							
Female	21	17	36.17	4	100.00	6.201	0.013
Male	30	30	63.83	0	0.00		

The proportion of low BMV patients was higher than high BMV in pre-obese (8.51% vs. 0.0%) and obese grade II (42.55% vs. 0.0%) while the proportion of high BMV was higher than low BMV in obese grade I (50.0% vs. 44.68%) and obese grade III (50.0% vs. 4.26%). Difference in BMV grade with different grades of obesity was found to be statistically significant (p=0.008) (Table 13).

**Table 13:** Association of bag mask ventilation (BMV) score with obesity grade χ2 - Chi square

Obesity Grade	Total	Low BMV Grade (n=47)		High BMV Grade (n=4)		Statistical Significance	
		No.	%	No.	%	χ^2^	p
Pre-obese	4	4	8.51	0	0.00	11.901	0.008
Obese Grade I	23	21	44.68	2	50.00		
Obese Grade II	20	20	42.55	0	0.00		
Obese Grade III	4	2	4.26	2	50.00		

The number of intubation attempts in the majority (n=42; 82.35%) patients was 1-2 attempts while in the rest nine (17.65%) patients was >3 attempts, and there was no failed intubation. The difference in number of intubation attempts among the patients of different age groups was not found to be statistically significant (p=0.469). Though the proportion of female patients with high intubation attempts was higher than those with low intubation attempts (66.67% vs. 35.71%) and vice versa for males, difference in number of attempts in male and female patients was not found to be statistically significant (p=0.087) (Table 14).

**Table 14:** Association of number of intubation attempts with demographic variables Low attempts: 1-2 attempts; High Attempts >3 attempts χ2 - chi square

Variables	Total	Low Attempts (n=42)		High Attempts (n=9)		Statistical Significance	
		No.	%	No.	%	χ^2^	p
Age Group (years)							
20-29	6	4	9.52	2	22.22	2.534	0.469
30-39	16	13	30.95	3	33.33		
40-49	22	18	42.86	4	44.44		
50-59	7	7	16.67	0	0.00		
Gender							
Female	21	15	35.71	6	66.67	2.932	0.087
Male	30	27	64.29	3	33.33		

Though the mean age, weight, and height of patients of low BMV grade were found to be higher than that of high BMV grade and vice versa for BMI, the difference in the mean value of the above variables of high BMV grade and low BMV grade patients was not found to be statistically significant (p>0.05). Mean values of all other variables except thyromental distance and hip circumference of high BMV grade patients were found to be higher than that of low BMV grade but the difference in mean values of patients with low CL grade and high CL grade was found to be statistically significant for neck circumference (40.30+3.43 vs 46.75+1.89 cm), thyromental distance (9.12+1.11 vs. 7.25+0.87 cm), NC/TMD ratio (6.48+0.96 vs. 4.43+0.68), skin fold thickness (30.86+3.70 vs 36.10+5.85 mm) and MP Grade (2.70+0.51 vs. 3.50+0.58) only (Table 15).

**Table 15:** Association of bag mask ventilation (BMV) score and patient characteristics * Statistical analysis performed using Mann-Whitney U test NC/TMD: neck circumference/thyromental distance ratio; W/H ratio: waist-hip ratio; MG grade: Mallampati grade

	Low BMV Grade (n=47)		High BMV Grade (n=4)		Statistical significance	
	Mean	SD	Mean	SD	't'/Z*	'p'
Age (yrs)	41.02	8.64	37.50	8.81	0.781	0.438
Weight (kg)	87.00	11.93	80.75	5.38	1.031	0.308
Height (mt)	1.56	0.13	1.48	0.10	1.270	0.210
BMI (kg/m^2^)	34.94	3.94	37.30	4.06	-1.145	0.258
Neck circumference (cm)	40.30	3.43	46.75	1.89	-3.691	0.001
Thyromental distance (cm)	9.12	1.11	7.25	0.87	3.271	0.002
NC/TMD ratio	4.43	0.68	6.48	0.96	-5.586	<0.001
Skin fold thickness (mm)	30.86	3.70	36.10	5.85	-2.605	0.012
Waist(cm)	102.34	10.06	102.50	1.00	-0.031	0.975
Hip (cm)	89.51	7.98	88.25	0.50	0.313	0.756
W/H ratio	1.14	0.06	1.15	0.01	-0.514	0.610
MP Grade*	2.70	0.51	3.50	0.58	-2.506	0.012

The proportion of patients with low intubation attempts was higher than high intubation attempts in pre-obese (9.52% vs. 0.0%), obese grade I (47.62% vs. 33.33%), and obese grade II (42.86% vs. 22.22%) while the proportion of high intubation attempts was higher than low intubation attempts in obese grade III (44.44% vs. 0.0%). Difference in the number of intubation attempts with different grades of obesity was found to be statistically significant (p<0.001) (Table 16).

**Table 16:** Association of number of intubation attempts with obesity grade χ2 - chi square

Obesity Grade	Total	Low Attempts (n=42)		High Attempts (n=9)		Statistical Significance	
		No.	%	No.	%	χ^2^	p
Pre-obese	4	4	9.52	0	0.00	20.664	<0.001
Obese Grade I	23	20	47.62	3	33.33		
Obese Grade II	20	18	42.86	2	22.22		
Obese Grade III	4	0	0.00	4	44.44		

Though the mean age and height of patients with low intubation attempts were found to be higher than that of high intubation attempts BMV grade and vice versa for weight and BMI, the difference in the mean value of the above variables except BMI (38.92+5.12 vs. 34.32+3.20 kg/m2) of high BMV grade and low BMV-grade patients was not found to be statistically significant (p>0.05). Mean values of all other variables except thyromental distance of high intubation attempt patients were found to be higher than that of low intubation attempts but the difference in mean values of patients with low intubation attempts and high intubation attempts was found to be statistically significant for neck circumference (39.69+2.46 vs 46.00+4.50 cm), thyromental distance (9.23+0.98 vs. 7.78+1.46 cm), NC/TMD ratio (4.30+0.57 vs. 5.92+0.91), skin fold thickness (30.69+3.81 vs 33.97+4.43 mm) and MP Grade (2.64+0.49 vs. 3.33+0.50) only (Table 17).

**Table 17:** Association of the number of intubation attempts and patient characteristics NC/TMD: neck circumference/thyromental distance ratio; W/H ratio: waist-hip ratio; MG grade: Mallampati grade

	Low Attempts (n=42)		High Attempts (n=9)		Statistical significance	
	Mean	SD	Mean	SD	't'/Z	'p'
Age (yrs)	41.38	8.57	37.78	8.74	1.141	0.259
Weight (kg)	85.45	11.21	91.44	13.04	-1.415	0.163
Height (mt)	1.56	0.13	1.54	0.10	0.328	0.744
BMI (kg/m^2^)	34.32	3.20	38.92	5.12	-3.498	0.001
Neck circumference (cm)	39.69	2.46	46.00	4.50	-5.935	<0.001
Thyromental distance (cm)	9.23	0.98	7.78	1.46	3.683	0.001
NC/TMD ratio	4.30	0.57	5.92	0.91	-6.875	<0.001
Skin fold thickness (mm)	30.69	3.81	33.97	4.43	-2.281	0.027
Waist(cm)	101.38	8.95	106.89	12.00	-1.576	0.121
Hip (cm)	89.02	7.53	91.22	8.45	-0.778	0.440
W/H ratio	1.13	0.06	1.16	0.05	-1.394	0.170
MP Grade	2.64	0.49	3.33	0.50	-3.181	0.001

The airway was found to be difficult in only four (7.84%) patients while the airway was easy in the rest 47 (92.16%) patients. Interestingly, these four patients were the same whose BMV score was of higher grade (>3), hence findings of high BMV were similar to those with difficult airways, and findings of low BMV grade were similar to those of easy airways. The difference in age of patients with difficult airway and easy airway (bag mask ventilation grade <3, Cormack-Lehane Grade <3 on direct laryngoscopy, the number of intubation attempts were <3.) was not found to be statistically significant (p=0.718). The proportion of females was higher in difficult airways (100.0%) as compared to easy airways (36.17%) and the proportion of males was higher in easy airways (63.83%) as compared to difficult airways (0.00%). The difference in the type of airway of males and females was found to be statistically significant (p=0.013) (Table 18).

**Table 18:** Association of difficult airway with demographic variables χ2 - chi square

Variables	Total	Easy Airway (n=47)		Difficult Airway (n=4)		Statistical Significance	
		No.	%	No.	%	χ^2^	p
Age Group (years)							
20-29	6	5	10.64	1	25.00	1.346	0.718
30-39	16	15	31.91	1	25.00		
40-49	22	20	42.55	2	50.00		
50-59	7	7	14.89	0	0.00		
Gender							
Female	21	17	36.17	4	100.00	6.201	0.013
Male	30	30	63.83	0	0.00		

The proportion of easy airway patients was higher than difficult airway in pre-obese (8.51% vs. 0.0%) and obese grade II (42.55% vs. 0.0%) while the proportion of difficult airway was higher than easy airway in obese grade I (50.0% Vs. 44.68%) and obese grade III (50.0% vs. 4.26%). Difference in the type of airway with different grades of obesity was found to be statistically significant (p=0.008) (Table 19).

**Table 19:** Association of difficult airway with obesity grade χ2 - chi square

Obesity Grade	Total	Easy Airway (n=47)		Difficult Airway (n=4)		Statistical Significance	
		No.	%	No.	%	χ^2^	p
Pre-obese	4	4	8.51	0	0.00	11.901	0.008
Obese Grade I	23	21	44.68	2	50.00		
Obese Grade II	20	20	42.55	0	0.00		
Obese Grade III	4	2	4.26	2	50.00		

Though the mean age, weight, and height of patients of easy airway grade were found to be higher than that of difficult airway grade and vice versa for BMI, the difference in the mean value of the above variables of difficult airway grade and easy airway grade patients was not found to be statistically significant (p>0.05).

Mean values of all other variables except thyromental distance and hip circumference of difficult airway grade patients were found to be higher than that of easy airway grade but the difference in mean values of patients with easy airway and difficult airway was found to be statistically significant for neck circumference (40.30+3.43 vs 46.75+1.89 cm), thyromental distance (9.12+1.11 vs. 7.25+0.87 cm), NC/TMD ratio (6.48+0.96 vs. 4.43+0.68) and skin fold thickness (30.86+3.70 vs 36.10+5.85 mm) only (Table 20).

**Table 20:** Association of difficult airway and patient characteristics *statistical analysis performed using Mann-Whitney U test. NC/TMD: neck circumference/thyromental distance ratio; W/H ratio: waist-hip ratio; MG grade: Mallampati grade

	Easy Airway Grade (n=47)		Difficult Airway Grade (n=4)		Statistical significance	
	Mean	SD	Mean	SD	't'/Z	'p'
Age (yrs)	41.02	8.64	37.50	8.81	0.781	0.438
Weight (kg)	87.00	11.93	80.75	5.38	1.031	0.308
Height (mt)	1.56	0.13	1.48	0.10	1.270	0.210
BMI (kg/m^2^)	34.94	3.94	37.30	4.06	-1.145	0.258
Neck circumference (cm)	40.30	3.43	46.75	1.89	-3.691	0.001
Thyromental distance (cm)	9.12	1.11	7.25	0.87	3.271	0.002
NC/TMD ratio	4.43	0.68	6.48	0.96	-5.586	<0.001
Skin fold thickness (mm)	30.86	3.70	36.10	5.85	-2.605	0.012
Waist(cm)	102.34	10.06	102.50	1.00	-0.031	0.975
Hip (cm)	89.51	7.98	88.25	0.50	0.313	0.756
W/H ratio	1.14	0.06	1.15	0.01	-0.514	0.610
MP Grade*	2.70	0.51	3.50	0.58	-2.506	0.012

Correlation of CL grade with NC/TMD ratio, BMV, no. of Intubation attempts, neck circumference, and inverse correlation with thyromental distance were found to be of moderate degree and statistically significant. The correlation of CL with MPG and difficult airway was mild and statistically significant; for the rest of the variables, the correlation was found to be weak/no correlation (Table 21).

**Table 21:** Correlation of patient characteristics with difficult airway. NC/TMD: neck circumference/thyromental distance ratio; W/H ratio: waist-hip ratio; MG grade: Mallampati grade; CL grade: Cormack-Lehane Grade

	CL		BMV		Intub. attempts		Difficult airway	
	r	p	r	p	r	p	r	p
Age (yrs)	0.21	0.143	0.15	0.282	-0.02	0.892	-0.09	0.522
Weight (kg)	0.13	0.349	-0.03	0.824	0.17	0.228	-0.15	0.304
Height (mt)	0.01	0.935	0.07	0.638	0.00	0.973	-0.20	0.153
BMI (kg/m^2^)	0.11	0.428	0.04	0.757	0.16	0.257	0.17	0.244
Neck circumference (cm)	0.57	0.000	0.48	0.000	0.62	0.000	0.40	0.004
Thyromental distance (cm)	-0.65	0.000	-0.70	0.000	-0.61	0.000	-0.38	0.006
NC/TMD ratio	0.76	0.000	0.74	0.000	0.77	0.000	0.44	0.001
Skin fold thickness (mm)	0.10	0.478	0.23	0.101	0.21	0.143	0.25	0.076
Waist(cm)	0.11	0.446	0.06	0.655	0.25	0.083	0.09	0.520
Hip (cm)	-0.03	0.821	-0.04	0.755	0.06	0.678	-0.03	0.849
W/H ratio	0.19	0.179	0.14	0.316	0.24	0.086	0.13	0.376
CL Grade	1.00	.	0.75	0.000	0.87	0.000	0.48	0.000
MPG	0.41	0.003	0.31	0.028	0.37	0.007	0.35	0.011
BMV	0.75	0.000	1.00	.	0.75	0.000	0.58	0.000
Intub. attempts	0.87	0.000	0.75	0.000	1.00	.	0.52	0.000
Difficult airway	0.48	0.000	0.58	0.000	0.52	0.000	1.00	.

Correlation of BMV grade with No. of Intubation attempts, CL grade, NC/TMD ratio, difficult airway, and inverse correlation with thyromental distance were found to be of moderate degree and statistically significant. The correlation of CL with MPG was mild and statistically significant; for the rest of the variables, the correlation was found to be weak/no correlation (Table 21).

The correlation of no. of intubation attempts with BMV grade, CL grade, NC/TMD ratio, difficult airway, and inverse correlation with thyromental distance was found to be of moderate degree and statistically significant. Correlation of No. of intubation attempts with MPG was mild and statistically significant; for the rest of the variables, the correlation was found to be weak/no correlation (Table 21)

The correlation of difficult airway with Intubation attempts and BMV was found to be of moderate degree and statistically significant. The correlation of difficult airway with MPG, NC/TMD, and CL grade was mild and statistically significant; for the rest of the variables, the correlation was found to be weak/no correlation (Table 21).

## Discussion

Demographic features

Among the demographic features, the patient’s sex showed a significant correlation with difficult airway. Among a total of 51 patients in our study, four patients who had difficulty with BMV and intubation were females. This is contradictory to study by Ezri T, et al. which showed higher incidence of difficult airway in males [10]. Whittle A, et al. have also observed a higher percentage of neck soft tissue (and fat) in obese males as compared to females. Age and ASA scores showed no correlation with difficult airways [11].

Obesity grade and BMI

Obese patients were classified initially on the basis of BMI cut-off values, proposed by a WHO expert committee for the classification of overweight, Seidell J. et al. similarly assessed obesity in their study using these cut off values [12]. As obesity has been found to be a risk factor for difficult airway, obesity grading in our study showed statistical significance. With difficult laryngoscopy (p=0.023), difficult intubation (p<0.001), difficult BMV (p=0.008), and hence difficult airway(p=0.008). However BMI failed to show any association with difficult airway in our study, which may be due to small sample size. This means that other factors associated with obesity might have an impact on airway difficulty like body fat distribution. A study conducted by Horner et al. also demonstrated that more fat was present in areas surrounding the collapsible segments of the pharynx in patients with OSA which may explain why some obese patients are easy to intubate or ventilate, while others are not [13].

Neck circumference

Neck circumference in obese patients has shown its statistical association with difficult airways. Recorded values of neck circumference in our study ranged from 35-49 cm (mean 40.8 cm) with higher values in the morbidly obese. Patients with difficult airways showed a mean value of 46.75 cm (p=0.001), Gonzalez H. et al. and Riad W. et al. identified NC values>43cm and >42cm, respectively, as an important predictor of difficult airway in their studies [14,15].

Thyromental distance (TMD)

Short TMD is one of the important clinical parameters used to predict a difficult intubation, although reports of the cut-off distance in the literature vary. In our study, this parameter showed high statistical significance with a difficult airway. The values recorded ranged between 6.0-11 cm with mean of 8.97 cm. A study done by Patil et al. concluded the normal value of the TMD in adults to be 6.5 cm or greater [16]. In our study, only three obese patients out of 51 patients had TMD of <6.5 cm and all of them were found difficult to intubate. These patients also had higher Mallampati scores. Similar observations were reported by Frerk C.M. which states that difficult intubation in patients with TMD less than 7 cm with poor visualisation of posterior pharyngeal wall during inspection of the oropharynx [17]. Tripathi M. et al. reported that patients with TMD greater than 4 cm could be easily intubated, as compared to TMD≤4 cm, tracheal intubation was found difficult in 48% of patients with Mallampati score is 1,2 and in 79% if the Mallampati score was 3 or 4 [18].

Neck circumference to thyromental distance ratio (NC/TMD)

This new predictor showed highly significant association with difficult airway in our study (p<0.001). Measured values ranged from 3.50-7.30 with a mean of 4.59. Patients with NC/TMD ≥ 5.5 showed airway difficulty. Kim W.H. et al. also observed in their study that ratio of the NC/TM≥5 is a better method than the previously reported Mallampati score or simple NC to predict difficult intubation for obese patients [19].

Mallampati score

Patients with Mallampati scores≥ 3 o r4 showed a statistically significant association with difficult mask ventilation, difficult laryngoscopy (p=0.014) and difficult intubation (p=0.001). Mallampati S.R. et al. also supported the hypothesis that difficult laryngeal visualisation can be predicted in most cases by eliciting the visibility of faucial pillars and uvula [7]. Juvin et al. stated association of higher Mallampati score with difficult intubation in obese patients; Brodsky J.B. et al. observed problematic intubation with increasing neck circumference and Mallampati score of ≥3; Srinivasa S. et al. found poor sensitivity, specificity, and negative predictive value of the Mallampati score and concluded that MPG should be combined with other airway assessment tests for prediction of difficult laryngoscopy or intubation [2,20,21].

Skin fold thickness

This new parameter was studied in our study to assess whether it can be employed as a tool to predict difficult airway in obese patients. Three readings were taken from the same site for each patient and their mean calculated to get a precise value. The minimum value recorded was 21.5 mm and the maximum was 42.5 mm with a mean of 31.27 mm. Surprisingly, the difference in mean values of patients with low intubation attempts to high intubation attempts and easy BMV to difficult BMV came out to be statistically significant (p=0.012 and 0.027 respectively), but it failed to show any significant correlation with difficult laryngoscopy and difficult intubation.

Other parameters

Weight, height, waist circumference, hip circumference, and waist-hip ratio (W/H) showed no statistically significant correlation with difficult laryngoscopy and difficult intubation in our study.

Bag and mask ventilation (BMV)

In the current study, the following observations were made. Firstly the reported incidence of difficult mask ventilation (DMV) was 7.84%. Studies done by Langeron O. et al. and MagalhãesI E. et al. reported an incidence of 5% and 16.3%, respectively [22,23]. These variations in incidence rate may be due to the lack of a standardized definition for DMV. Secondly, the patients with DMV had difficult laryngoscopy and difficult intubation. A similar association was seen in studies conducted by Riad W. et al. and Langeron O et al. [15,22]. Thirdly, obesity grade and female sex were associated with DMV. Juvin et al., Kim et al., and Langeron O. et al. observed in their studies that higher incidence of difficult face mask ventilation in obese patients which may be due to fat tissue deposition in the hypo-pharynx, uvula, tongue, and arytenoid folds, increasing the volume of these structures and reducing the free area for air passage rather than sniffing position [2,19,22]. As we adopted the sniff position initially for all subjects, the incidence of difficult intubation may have been increased for obese patients. Fourthly, OSA, one of the important predictors of difficult airways in obese, was not included in the study. And lastly, the definition of DMV was subjective in our study. However, the anaesthesiologist was considered as an expert able to recognize the occurrence of a clinically relevant DMV.

Intubation

The effect of obesity on difficulty of intubation and the utility of available single predictive indices is unclear, as suggested in previous studies conducted by Shiga T. et al. and Lee A. et al. [24,25]. While El-Ganzouri A. et al. and Cortellazzi P. et al. concluded in their studies that a combination of individual tests or risk factors adds to the incremental diagnostic value in comparison with the value of each test alone [26,27].

The incidence of difficult airway was 7.84% in our study, however in previous studies by Juvin P. et al. and Kim W. H. et al., showed incidence of 15.5% & 13.8%, respectively [2,19]. The reason for this lower incidence in our study may be a small study sample size.

Limitations

Since the KGMU is a teaching institute, laryngoscopy was performed by the resident anaesthesiologist (supervised by a qualified anaesthesiologist) scheduled for that procedure. This resident may have been in the first, second, or third year of the residency program. The classification was then confirmed by the chief anaesthesiologist. In cases of discordance, the classification suggested by the chief anaesthesiologist was noted finally. Although our study was not blinded, the patient’s initial position may have influenced the incidence of difficult intubation. Due to small size of sample, correlation between predictors of obesity including BMI and Difficult airway is lacking in our study, although few studies demonstrated that BMI have a well built association with difficult airway and difficult tracheal intubation [ 28,29]. A larger sample size will be required to prove our hypothesis that skin fold thickness and other predictors of obesity can be a useful test for pre‑operative airway assessment in obese patients.

## Conclusions

Neck circumference (NC), thyromental distance (TMD), and neck circumference to thyromental distance ratio (NC/TMD) depicted high statistical significance and close association with airway difficulty in obese patients. Obesity grade is a risk factor for difficult airway, but predictors of obesity like skin fold thickness, waist-hip ratio, etc. individually did not show association with difficult laryngoscopy, difficult intubation, and difficult BMV in our study (small sample size may be a limiting factor). A high Mallampati score is a risk factor in obese patients. Bag mask ventilation difficulty in obese is strongly associated with intubation difficulty. Female patients only showed difficult airways among the obese study sample. Our results are consistent with previous studies, which have demonstrated that none of the commonly performed tests alone has proven to be adequate in predicting difficult intubation in the obese population. However, a small sample size was a limitation in our study and a large sample size will be required to prove the hypothesis of association and correlation of predictors of obesity with difficult airway individually.
